# 肺癌术前常规纵隔淋巴结外科分期的临床价值

**DOI:** 10.3779/j.issn.1009-3419.2010.06.011

**Published:** 2010-06-20

**Authors:** 烨 徐, 阳 申屠, 敏 郑, 明 郭

**Affiliations:** 1 200336 上海，上海市长宁区中心医院胸外科 Department of Thoracic Surgery, Shanghai Changning District Central Hospital, Shanghai 200336, China; 2 200030 上海，上海市胸科医院/上海市肺部肿瘤临床医学中心胸外科 Shanghai Chest Hospital/Shanghai Lung Tumor Clinical Medical Center, Shanghai 200330, China

**Keywords:** 肺肿瘤, 分期, 计算机断层显像, 纵隔镜检查术, Lung neoplasms, Stage, Computer tomography, Mediastinoscopy

## Abstract

**背景与目的:**

探讨肺癌术前常规纵隔淋巴结外科分期的临床价值。

**方法:**

76例肺癌患者开胸术前常规行纵隔淋巴结活检，以术后病理为金标准，比对术前胸部CT和纵隔镜对肺癌纵隔淋巴结转移的诊断效能。

**结果:**

术前胸部CT对纵隔淋巴结转移的诊断敏感性、特异性、准确性、阳性预测值和阴性预测值分别为68.5%、66.7%、68.4%、84.6%和16.7%。纵隔镜检查术则分别为87.5%、100%、84.2%、100%和60%。

**结论:**

肺癌术前常规纵隔镜检查术对纵隔淋巴结分期的优势明显，具有极高的临床实用价值。

## Abstract (en)

**Background and objective:**

The aim of this study is the clinical value of preoperative routine surgical staging to mediastinal lymph nodes on lung cancer.

**Methods:**

Seventy-six cases underwent lymph nodes biopsy with mediastinoscopy. The diagnostic efficacy of thoracic CT scan and mediastinoscopy on mediastinal lymph nodes metastasis were compared.

**Results:**

The sensitivity, specificity, accuracy, positive predictive value and negative predictive value of thoracic CT scan and mediastinoscopy on diagnosing mediastinal lymph nodes metastasis were 68.5%, 66.7%, 68.4%, 84.6%, 16.7% and 87.5%, 100%, 84.2%, 100%, 60%, respectively.

**Conclusion:**

Routine preoperative mediastinoscopy had obvious advantage compared with thoracic CT scan on mediastinal lymph nodes staging. The routine preoperative surgical staging of mediastinal lymph nodes on lung cancer had high clinical value.

研究^[1]^显示，原发性支气管肺癌（以下简称肺癌）发生纵隔淋巴结转移（N2）患者的5年生存率远低于N0、N1者。我国肺癌患者术后5年生存率一直徘徊在40%左右^[2]^，远低于发达国家。分析表明，其中最重要的原因在于我国手术病例中Ⅲ期患者的比例过高（超过40%）^[3]^。最新的《新英格兰医学杂志》甚至将N2的手术列为无效开胸手术^[4]^。可见准确的术前分期攸关治疗方案的确立和预后。目前，术前判断肺癌纵隔淋巴结转移的实际方法仍主要是胸部计算机断层扫描（computer tomography, CT），但其准确性一直受到质疑。近年来，术前纵隔淋巴结的外科分期价值日益显现，但国内现状依然是理念落后，普及不力。我院近年开展纵隔镜技术，本文拟比较研究术前CT和常规纵隔镜检查术对纵隔淋巴结转移的诊断效能和临床价值。

## 1 资料与方法

### 1.1 病例资料

2005年12月-2009年10月，上海市长宁区中心医院联合上海市胸科医院共对76例非小细胞肺癌（non-small cell lung cancer, NSCLC）患者行开胸前电视纵隔镜检查术。其中男性48例，女性28例。患者年龄45岁-69岁，中位年龄53岁。

### 1.2 诊治程序

所有患者入院后依照常规术前准备，并统一行胸部增强CT，根据通行的判断标准，由专业CT医师对患者纵隔淋巴结作出临床分期。手术当日，对患者先行纵隔镜检查术以活检纵隔淋巴结，随后开胸行规范肺叶切除并系统性淋巴结清扫，达到肺癌完全性切除标准^[5]^。

### 1.3 胸部CT分期方法

术前测量增强胸部CT各组纵隔淋巴结最短径，设定 < 1 cm为阴性（无转移），1 cm-1.5 cm为可疑转移，>1.5 cm为阳性（转移）。据此判断有无转移，获得每一病例术前纵隔淋巴结的临床分期。

### 1.4 纵隔镜检查术方法

患者取仰卧位，使用德国Karl Storz 20131520型电视纵隔镜，所有患者均经颈活检双侧#2组、#3组、#4组及#7组纵隔淋巴结，左肺患者经左胸第2前肋间加行#5组、#6组纵隔淋巴结活检。

### 1.5 纵隔淋巴结清扫范围

右侧开胸患者清扫#2R组、#3R组、#4R组、#7组、#8R组、#9R组纵隔淋巴结，左侧开胸患者清扫#5组、#6组、#7组、#8L组、#9L组纵隔淋巴结。

### 1.6 病理检查方法

所有手术标本均送常规病理检查，HE染色，光学显微镜观察。获得：①纵隔镜活检的纵隔淋巴结（#2R组、#3R组、#4R组、#5组、#6组、#7组）病理；②系统性清扫的纵隔淋巴结（#2R组、#3R组、#4R组、#5组、#6组、#7组、#8L组、#9L组）病理；③患者术后病理分期。

### 1.7 统计学方法

以患者术后纵隔淋巴结病理检查结果为“金标准”，分别列出纵隔淋巴结转移的诊断试验四格表，计算胸部增强CT和纵隔镜检查术之诊断敏感性、特异性、准确性、阳性预测值和阴性预测值等指标。

## 2 结果

### 2.1 临床治疗结局

本组76例NSCLC患者均行肺叶切除及系统性纵隔淋巴结清扫。术后1例并发肺部感染，继而肺功能不全导致死亡，余75例患者临床痊愈出院，具体手术方式的病例数分布详见表 1。

**1:** 患者手术方式的病例数 The case number of operation style

Operation style					
RUL	RML	RLL	LUL	LLL	Total
33	2	10	25	6	76
RUL: right upper lobectomy; RML: right middle lobectomy; RLL: right lower lobectomy; LUL: left upper lobectomy; LLL: left lower lobectomy.					

### 2.2 分期数据

开胸术前CT、纵隔镜及术后病理分期的具体数据详见表 2。

**2:** 不同分期方法的病例数分布 The case number of different staging methods

Stage	Ⅰa	Ⅰb	Ⅱa	Ⅱb	Ⅲa	Ⅲb	Ⅳ	Total
CT	7	25	3	10	24	7	0	76
Mediastinoscopy	5	15	3	5	34	14	0	76
Pathology	3	9	3	9	37	15	0	76
CT: computer tomography.								

### 2.3 CT及纵隔镜对纵隔淋巴结转移的诊断评价

术前胸部CT诊断纵隔淋巴结阳性48例，经手术证实44例；诊断纵隔淋巴结阴性28例，经手术证实20例。纵隔镜诊断纵隔淋巴结阳性56例，与手术清扫的病理结果完全一致；纵隔镜诊断纵隔淋巴结阴性的20例中，有8例手术清扫证实阳性。检查结果见表 3，诊断效能评价见表 4。

**3:** CT及纵隔镜对纵隔淋巴结转移的诊断试验结果 The diagnostic test results of CT scan and mediastinoscopy on mediastinal lymph node metastasis

		Pathology					Pathology		
		+	-	Total			+	-	Total
CT	+	44	4	48	Mediastinoscopy	+	56	0	56
	-	8	20	28		-	8	12	20
Total		52	24	76	Total		64	12	76

**4:** CT及纵隔镜对纵隔淋巴结转移的诊断评价 The diagnostic evaluation of CT scan and mediastinoscopy on mediastinal lymph node metastasis

	CT	Mediastinoscopy
Sensitivity	68.5%	87.5%
Specificity	66.7%	100%
Accuracy	68.4%	84.2%
Positive predictive value	84.6%	100%
Negaitive predictive value	16.7%	60%

## 3 讨论

大样本的回顾研究^[1]^显示，NSCLC手术后的5年生存率，Ⅰa期为67%，Ⅰb期57%，Ⅱa期55%，Ⅱb期39%，Ⅲa（N2）期仅23%。众所周知，Ⅰ期和Ⅱ期NSCLC能够完全手术切除，Ⅲa（N2）期NSCLC的手术指证仍存争议。研究^[6]^显示，对于Ⅲa期，仅有单个小淋巴结转移的微小淋巴结病变可以完全切除，但这种情况仅占Ⅲa期病例总数的四分之一，而其5年生存率可高达41.6%^[7]^。因此，术前准确筛检出可手术的Ⅰ期、Ⅱ期和部分Ⅲa期（单站N2）病例就成为NSCLC分期的主要任务，此举对于确认NSCLC的手术价值、提高患者术后生存率具有十分重要的意义。

现行的肺癌分期主要包括临床分期（cTNMcStage）、病理分期（pTNM-pStage）和再治疗分期（rTNM-rStage），此外尚有不成熟的分子分期（mTNMmStage）。目前，肺癌的治疗前临床分期仍然主要依靠影像学信息，在中国，胸部CT是预测肺癌纵隔淋巴结转移的最主要的影像方法。CT通常以淋巴结的大小来判断有无转移，但Kerr在1992年的报道即指出^[8]^：不同直径淋巴结的肿瘤转移率均在20%左右，纵隔淋巴结大小与转移间无统计学意义。Toloza^[9]^的*meta*分析显示，CT对纵隔淋巴结转移评估总的敏感性为57%，特异性为82%，阳性预测值为56%，阴性预测值为83%。中国的资料：1组258例肺癌、2 892枚纵隔淋巴结的研究表明：无转移淋巴结的平均直径（7.05 mm）小于转移淋巴结的平均直径（10.70 mm），*P* < 0.005，但问题是有44.3%的转移淋巴结直径 < 10 mm，而在无转移淋巴结中72.9%至少有1枚的直径>10 mm，结论是淋巴结的大小不能作为非小细胞肺癌淋巴结转移的可靠参数；CT对肺癌纵隔淋巴结转移的诊断敏感性为57%，特异性80.2%^[10]^。国外报道的临床分期与病理分期吻合率只有35.1%-45.4%^[11]^，意味着影像学（CT）对将近一半以上NSCLC患者的术前分期并不准确。一般研究中，大多以淋巴结短径>1 cm作为正常和异常间的界限，考虑到淋巴结肿大愈明显，转移机率愈高的一般规律，本组以淋巴结短径>1.5 cm作为界限值，期望更严格标准藉以提高CT的诊断水平。但即便如此，其诊断敏感性、特异性及准确性仍未超过70%，此举虽使阳性预测值有所提高（84.6%），却降低了阴性预测值（16.7%）。从表 2看，本组术前CT评估Ⅰa-Ⅱb的比例几近60%（45/76），实际病理不到32%（24/76），明显高估早期可手术病例；对Ⅲa、Ⅲb的预测（31/76, 40.8%）则明显低于病理结果（52/76, 68.4%），呈低估晚期态势。由此，我们同样认为，CT对NSCLC术前纵隔淋巴结转移的判断价值有限。

纵隔镜检查术是目前应用最广泛的纵隔淋巴结外科分期手段，是迄今评估纵隔淋巴结状况最准确的手段^[12]^。Pearson大样本的临床实践^[13]^表明，纵隔镜对纵隔淋巴结转移的诊断敏感性、特异性、准确性、阳性预测值和阴性预测值分别为96%、100%、95%、100%和93%，具有极高的诊断效能，以至于有作者认为该技术可作为肺癌术前纵隔淋巴结分期的金标准^[14]^。本组的诊断指标亦达到较满意水平，明显优于胸部CT，有4例纵隔镜检查假阴性，纵隔淋巴结直径均 < 1 cm，活检不易取材，操作经验有待积累。鉴于肺癌分期治疗的重要性，北美和欧洲十分重视术前的准确分期，因而纵隔镜检查术得以广泛应用。在国内，部分医患主观理念上对肺癌术前分期的意义认识不足，加之纵隔镜设备的匮乏、手术潜在的高风险、病理结果的假阴性以及不菲的手术价格等客观因素，导致外科分期的接受度极低，限制了该技术的广泛而常规的临床应用。在临床实践中，肺癌术前纵隔镜检查的应用指证一直是学术界争论的焦点。通常认为以下情形需行纵隔镜检查：CT下纵隔淋巴结>1 cm者、中央型肺癌、分化差的肿瘤、新辅助治疗与治疗后评估、T3、T4肿瘤判断纵隔淋巴结转移的水平和数目以决定是否手术治疗。但有研究^[15]^显示，即使CT下纵隔淋巴结无肿大（< 1 cm）的Ⅰ期病变仍然需要进行纵隔镜检查，因为Ⅰ期病变纵隔淋巴结隐性转移的发生率可达6.9%-11.1%。但基于较高的手术费用及风险，作为肺癌术前常规检查，医患双方的顾虑完全可以理解。Esnaola等^[16]^利用决策树分析法比较了CT、选择性纵隔镜检查、常规纵隔镜3种术前分期策略，结果显示，术前接受常规纵隔镜检查的NSCLC患者获得了最大的质量调整预期寿命，并使N2、N3患者避免无益的手术，使N0、N1患者避免术前不必要的新辅助治疗。对T1患者，常规纵隔镜检查的生存效益高于选择性纵隔镜，但基于经济学观点，临床T1患者应用选择性纵隔镜检查较为适宜。本研究显示，术前常规纵隔镜检查评估Ⅰa-Ⅱb的比例为36.80%（24/76），与实际病理完全一致，对Ⅲa、Ⅲb的预测（48/76, 63.2%）亦较接近病理结果（52/76, 68.4%），由此，我们倾向于支持术前常规纵隔镜检查在NSCLC的应用。

纵隔镜检查术对纵隔淋巴结转移的评估准确可靠，对适宜手术病例的选择具有实际指导价值，可作为肺癌术前的常规检查应用于临床。
